# Targeting Th17 Cells with Small Molecules and Small Interference RNA

**DOI:** 10.1155/2015/290657

**Published:** 2015-12-17

**Authors:** Hui Lin, Pingfang Song, Yi Zhao, Li-Jia Xue, Yi Liu, Cong-Qiu Chu

**Affiliations:** ^1^Department of Rheumatology and Immunology, West China Hospital, Sichuan University, Chengdu, Sichuan 610041, China; ^2^Division of Arthritis and Rheumatic Diseases, Oregon Health & Science University and VA Portland Health Care System, Portland, OR 97239, USA

## Abstract

T helper 17 (Th17) cells play a central role in inflammatory and autoimmune diseases via the production of proinflammatory cytokines interleukin- (IL-) 17, IL-17F, and IL-22. Anti-IL-17 monoclonal antibodies show potent efficacy in psoriasis but poor effect in rheumatoid arthritis (RA) and Crohn's disease. Alternative agents targeting Th17 cells may be a better way to inhibit the development and function of Th17 cells than antibodies of blocking a single effector cytokine. Retinoic acid-related orphan receptor gamma t (ROR*γ*t) which acts as the master transcription factor of Th17 differentiation has been an attractive pharmacologic target for the treatment of Th17-mediated autoimmune disease. Recent progress in technology of chemical screen and engineering nucleic acid enable two new classes of therapeutics targeting ROR*γ*t. Chemical screen technology identified several small molecule specific inhibitors of ROR*γ*t from a small molecule library. Systematic evolution of ligands by exponential enrichment (SELEX) technology enabled target specific aptamers to be isolated from a random sequence oligonucleotide library. In this review, we highlight the development and therapeutic potential of small molecules inhibiting Th17 cells by targeting ROR*γ*t and aptamer mediated CD4^+^ T cell specific delivery of small interference RNA against ROR*γ*t gene expression to inhibit pathogenic effector functions of Th17 lineage.

## 1. Introduction

The differentiation of naïve CD4^+^ T cells into effector T helper (Th) cells is induced by their T cell receptor and costimulatory molecules in the presence of other cytokines. It is that these cytokines and transcriptional factors ultimately determine the differentiation of CD4^+^ Th cells into distinct subsets. Initially, CD4^+^ Th cells were identified as having two subsets, Th1 and Th2 cells [1]. Th1 cells produce high levels of IFN-*γ* and express the transcriptional factor T-bet, which protect the host against intracellular pathogens [2]. Th2 cells express GATA-3 and produce IL-4, IL-5, IL-9, and IL-13 which are mainly involved in protection against parasitic helminthes [3]. Recently, new subsets of effector Th cells that express different transcriptional factors and produce distinct cytokines have been discovered, including T regulatory (Treg) cells, Th17 cells, follicular helper T cell (Tfh), and Th9 cells [4, 5]. Treg cells are characterized by the production of IL-10 and TGF-*β* as major cytokines and expression of forkhead box P3 (Foxp3) as transcriptional factor, which control immune response and maintain immune tolerance [6]. Th17 cells are characterized by the production of IL-17A (also known as IL-17), IL-17F, and IL-22 as signature cytokines and expression of retinoic acid-related orphan receptor gamma t (ROR*γ*t) as master transcriptional factor [7–9]. These cytokines play a critical role in host defense against extracellular pathogens such as bacteria and fungi [10] and many autoimmune diseases, including psoriasis, rheumatoid arthritis (RA), inflammatory bowel disease, uveitis, and multiple sclerosis [11–13]. Fully human monoclonal antibodies (mAbs) against IL-17 (ixekizumab and secukinumab) and IL-17 receptor A (IL-17RA) (brodalumab) have rapidly reduced clinical symptoms in patients with psoriasis [14–18]. However, in a Phase IB study on methotrexate-resistant RA patients, brodalumab did not improve disease symptoms [19]. In a Phase II study, secukinumab did not show clinical efficacy in RA patient with inadequate response to methotrexate [20]. Furthermore, treatment of patients with Crohn's disease with secukinumab not only showed no good responses, but also worsened disease in some patients [21]. These data suggest that targeting IL-17 cannot completely alleviate Th17-mediated autoimmune diseases. Since Th17 cells also produce other cytokines such as IL-17F and IL-22 which are potent inflammatory mediators, targeting Th17 cells may provide a better efficacy in these clinical conditions [22].

Th17 differentiation requires the master transcriptional factor, ROR*γ*t, which is induced by activation of naïve CD4^+^ T cells in the presence of inflammatory cytokines, such as IL-6, TGF-*β*, IL-21, IL-1*β*, and IL-23 [23]. Mice deficient in ROR*γ*t have reduced Th17 differentiation and are resistant to experimental autoimmune encephalomyelitis (EAE) [9]. Conversely, overexpression of ROR*γ*t promotes IL-17 production. The critical role of ROR*γ*t in the generation of Th17 cells provides a unique opportunity to develop novel therapeutics targeting Th17 cells. Given the fact that cytokines of IL-17 family are important in host defense and they are also produced by other immune cells other than Th17 cells, it is highly desirable to target the pathogenic Th17 cells. The disadvantage of mAbs targeting individual IL-17 cytokines is that it does not discriminate the cellular source of IL-17 and therefore poses potential adverse effects from blocking IL-17 activity produced for host defense. Moreover, the effector cytokines of Th17 cells include IL-17A, IL-17F, and IL-22 which are all to be blocked to abrogate Th17 cell activity which is a challenging task for individual mAbs.

Recently, small molecules targeting ROR*γ*t have been identified, which not only suppress Th17 differentiation and IL-17 production, but also reduce the severity of animal models of autoimmune diseases. In addition, recent advancement in technology of engineering nucleic acid enables a targeted delivery of small interference RNA (siRNA) or short hairpin RNA (shRNA) using aptamers which serve as vehicle to guide siRNA or shRNA to target cells. These two classes of agents, which are nonmonoclonal antibody or fusion protein based, are emerging to be useful in targeting Th17 cells rather than merely blocking individual cytokines. Small molecules directly interact with ROR*γ*t to block its activity while siRNA/shRNA specifically inhibits ROR*γ*t gene expression.

## 2. Identification and Differentiation of Th17 Cells

Before the identification of Th17 cells, T cell mediated autoimmunity was believed to be mediated by Th1 cells. Indeed, T-bet deficient mice were resistant to EAE, and polyclonal antibody targeting IL-12 was an effective therapy for EAE and CIA. However, the later studies provided contradictory results that IFN-*γ* and IFN-*γ* receptor deficient mice, as well as mice that lack IL-12p35, were not protected from EAE but developed rapidly progressing disease [24, 25]. Furthermore, IFN-*γ* knockout mice develop severe EAE and convert resistant strain of mice to be highly susceptible to collagen-induced arthritis (CIA) [26, 27]. Thus, the function of Th1 cells in T cell mediated autoimmunity was challenged. The discovery of IL-23, a cytokine which is composed of a unique p19 subunit and a p40 subunit which is shared with IL-12 [28], provided us with novel insights. It was IL-23, not IL-12, that was critical for the induction of EAE and CIA [29, 30]. Moreover, IL-23 failed to induce IFN-*γ* but instead expanded IL-17-producing T cells. When IL-17-producing T cells induced by IL-23 were adoptively transferred into naïve wild-type mice, EAE developed [30]. IL-23p19-deficient mice were resistant to EAE due to lack of IL-17-producing T cells [29, 30]. These studies led to IL-17-producing T cells to be described as a distinct Th cell subset, which was named Th17 cells [7, 8].

Differentiation of Th17 cells is induced by activation of naïve CD4^+^ T cells in the presence of inflammatory cytokines. Transforming growth factor- (TGF-) *β* is a regulatory cytokine which has multiple effects on T cell development, homeostasis, and tolerance [31]. TGF-*β* not only induces naïve precursors into Foxp3-expressing inducible Treg (iTreg) [30], but also plays a crucial role in the generation of Th17 [31]. However, TGF-*β* alone is not capable of the induction of Th17 cells development. Unlike Th1, Th2, and iTreg cells, which only require a single cytokine for their generation, additional differentiation factors are required in Th17 cells development. Recent studies found that combination of IL-6 and TGF-*β* was the essential cytokine-mix of inducing naïve T cells to develop Th17 cells [32–34]. IL-6 is able to inhibit TGF-*β*-driven induction of Foxp3 in naïve T cells and instead leads to strong induction of IL-17 [33]. Furthermore, IL-21 together with TGF-*β* is also able to induce the differentiation of Th17 cells. During the initial Th17 differentiation, IL-6 induced IL-21 acting as a positive amplification loop to enforce Th17 differentiation [35, 36]. IL-21 was shown to be able to replace IL-6 at least* in vitro* [37]. In the absence of IL-6, IL-21 together with TGF-*β* was able to inhibit the development of iTreg and to promote the differentiation of Th17 cells [37].* In vivo*, however, the role of IL-21 in the induction of Th17 cells remains controversial. It had been reported that the absence of IL-21 or IL-21R had no significant difference on the development of Th17 cells [38, 39]. Thus, IL-21 might be an alternative pathway in inducing and expanding Th17 cells [23]. IL-23 also plays an important role in regulation of Th17 cells indirectly. However, IL-23 receptors are absent on naïve T cells, so IL-23 is not involved in the initiation of Th17 cells, but expands an existing population of effector Th17 cells [40]. Without IL-23, activated CD4^+^ T cells in the presence of IL-6 plus TGF-*β* were able to produce high amounts of IL-17 but did not fully develop into pathogenic Th17 cells [41]. The treatment with neutralizing IL-23p19 specific antibody not only inhibited the development of EAE but also ameliorated EAE after the onset of disease [42]. Ustekinumab, a mAb against IL-23/IL-12p40, has shown a marked efficacy in clinical studies involving psoriasis patients [43]. Ustekinumab also has shown increased clinical responses in patient with tumor necrosis factor- (TNF-) refractory Crohn's disease [44]. These studies indicate that IL-23 is an important cytokine in Th17-mediated autoimmune disease. In contrast to mice, combination of IL-6 and TGF-*β* is not capable of inducing human Th17 differentiation [45]. Instead of TGF-*β*, IL-1*β* together with IL-6 or IL-23 was reported to upregulate ROR*γ*t and induce IL-17 production from CD4^+^ T cells isolated from human peripheral blood, suggesting a fundamental difference in the biology of human and mouse Th17 cells [46].

## 3. Transcriptional Regulation of Th17 Cells

The differentiation of Th17 cells is initiated by the combined signals of activated TCR and cytokine receptors. These signals then induce specific transcription factors responsible for the expression of Th17 cell specific genes such as* Il17* and* Il17f*. Multiple transcription factors have been shown to be important for the development of Th17 cells, including ROR*γ*t, STAT3, IRF4, BATF, and RUNX1. ROR*γ*t is the master transcription factor that regulates the differentiation of Th17 cells [47]. ROR*γ*t belongs to the ROR subfamily. ROR is the member of retinoic acid nuclear receptor superfamily containing a ligand-binding domain (LBD). Usually, ligand binding to the LBD of ROR leads to conformational change and transcriptional activity. The ROR subfamily has three members in mammals: ROR*α*, ROR*β*, and ROR*γ* [48]. The ROR*γ* has two different isoforms: ROR*γ* and ROR*γ*t, which are encoded by the* Rorc* gene and have difference only at their N terminus [49]. ROR*γ*t is a splice variant of ROR*γ* expressed in T cells [49]. Unlike ROR*γ*, which is expressed in many tissue such as heart, kidney, liver, lung, brain, and muscle, ROR*γ*t is expressed exclusively in lymphoid cells [50]. ROR*γ*t is an important molecule to regulate gene expression during the development of T cells and the formation of secondary lymphoid organ [51–53].* Rorc* gene knockout mice exhibited that CD4^+^CD8^+^ thymocytes showed early apoptosis, and lymph nodes, Peyer's patches, and lymphoid tissue inducer (LTi) cells failed to develop [52, 53].* In vitro*, with the absence of* Rorc* in CD4^+^ T cells, IL-17 expression was greatly decreased under Th17 polarizing conditions. Conversely, overexpression of ROR*γ*t in naïve CD4^+^ T cells was sufficient to induce the expression of IL-17, IL-17F, and IL-22 [9]. ROR*γ*t is necessary for the expression of IL-17 as well as the differentiation of Th17 in mouse and human CD4^+^ T cells [9, 54]. The number of Th17 cells was markedly reduced and the disease severity of EAE alleviated in* Rorc*-deficient mice. The role of ROR*γ*t is similar to transcription factors such as T-bet and GATA3 in Th1 and Th2 differentiation, respectively, and therefore ROR*γ*t has been considered to be a “master transcriptional factor” for Th17 differentiation [47]. ROR*γ*t promotes IL-17 expression by directly binding the promoter region of* Il17* gene at multiple sites [9, 55, 56].

Another related retinoic acid nuclear receptor, ROR*α*, is also expressed in Th17 cells both* in vitro* and* in vivo*. In contrast to ROR*γ*t, ROR*α* played minimal roles in mouse Th17 differentiation. However, mice deficiencies in* Rora* and* Rorc* markedly impaired Th17 generation and completely protected mice from EAE [57]. The coexpression of* Rora* and* Rorc* induced greater Th17 differentiation. It is demonstrated that ROR*α* and ROR*γ*t acts as synergy in regulating Th17 cell gene expression.

Besides ROR*α* and ROR*γ*t, other transcription factors are required in Th17 differentiation. The transcription factor signal transducer and activator of transcription 3 (STAT3), which is preferentially activated by IL-6, IL-21, and IL-23, is capable of inducing ROR*γ*t and regulating Th17 cells development [58, 59]. Deficiency of STAT3 in CD4^+^ T cells impaired Th17 differentiation* in vivo*, and overexpression of a constitutively active STAT3 could increase IL-17 production [58, 60]. STAT3 might affect the production of IL-17 by increasing the expression of ROR*γ*t and ROR*α* [57, 58]. Furthermore, STAT3 also binds directly to the* Il17* and* Il21* promoters and leads to the expression of IL-17 and IL-21 [61, 62]. Therefore, STAT3 and ROR*γ*t seem to cooperate to induce IL-17 production. Transcription factor interferon regulatory factor 4 (IRF4) also has a certain role in Th17 differentiation, which was previously associated with GATA-3 expression in Th2 differentiation [63]. Recently, it has been shown that IRF4 regulates IL-17 and IL-21 production [64]. IRF-4 deficient mice were shown to impair Th17 responses and were resistant to EAE [65]. IRF-4 deficient T cells failed to upregulate ROR*γ*t in response to IL-6 plus TGF-*β* and did not differentiate into Th17 cells [65], suggesting that IRF4 might also cooperate with ROR*γ*t to induce Th17 differentiation. In addition, BATF, a member of the AP-1 transcription factor family, and Runx1, a member of RUNX1 transcription factor, are also important for Th17 differentiation [66, 67].

As mentioned above, ROR*γ*t, STAT3, IRF4, BATF, and RUNX1-deficient mice show an impaired Th17 generation and an attenuated susceptibility to the induction of autoimmunity. Targeting these transcription factors might be a possible way to inhibit the development and function of Th17 cells. ROR*γ*t acts as the master transcription factor of Th17 differentiation, resulting in an attractive pharmacologic target for the treatment of Th17-mediated autoimmune disorders.

## 4. Small Molecules Target to Th17 Cells

### 4.1. Digoxin

By performing a chemical screen with an insect cell-based reporter assay, the cardiac glycoside digoxin was identified as a specific inhibitor of ROR*γ*t transcriptional activity. Digoxin suppressed murine Th17 cell differentiation without affecting differentiation of other T cell lineages. In addition, digoxin was effective in attenuating EAE in mice and in delaying the onset and reducing disease severity in a rat model of adjuvant-induced arthritis [68–70]. Digoxin was toxic for human cells at high doses, but its synthetic derivatives 20,22-dihydrodigoxin-21,23-diol and digoxin-21-salicylidene were nontoxic and specifically inhibited the induction of IL-17 in human CD4^+^ T cells [68]. These data indicate that derivatives of digoxin might be used as chemical templates for the development of targeting ROR*γ*t therapeutic agents that attenuate inflammatory Th17 cells function and autoimmune disease.

### 4.2. ML209/Compound 4n

Using a cell-based gene ROR*γ*t and control reporter assay, a small molecule library comprising 300,000 compounds was screened at the NIH Chemical Genomics Center (NCGC), a series of diphenylpropanamide compounds as a selective ROR*γ*t inhibitor, including a highly potent compound ML209 (also known as compound 4n). Huh and colleagues found that compound 4n inhibited transcriptional activity of ROR*γ*t, but not ROR*α*, in cells. Like digoxin, compound 4n selectively inhibited murine Th17 differentiation without affecting the differentiation of naïve CD4^+^ T cells into other lineages, including Th1 and regulatory T cells. Moreover, compound 4n suppressed ROR*γ*t-induced expression of IL-17 in human T cells [71]. This report demonstrates that compound 4n might serve as a valuable pharmacological agent to inhibit ROR*γ*t transcriptional activity and Th17 differentiation.

### 4.3. SR1001 and SR2211

Using the liver X receptor (LXR) agonist T0901317 [72] scaffold as a lead compound, Griffin and Burris developed a derivative, SR1001, which was devoid of all LXR activity yet retained its ability to suppress the transcriptional activity of ROR*α* and ROR*γ* [73]. SR1001 not only is high-affinity synthetic ligand that is specific to both ROR*α* and ROR*γ*, but also inhibits Th17 cell differentiation and function. SR1001 binds specifically to the LBD of ROR*α* and ROR*γ*, inducing a conformational change within the LBD, resulting in suppression of the receptors' transcriptional activity. By suppressing IL-17 gene expression and protein production, SR1001 inhibited the development of murine Th17 cells. Furthermore, SR1001 inhibited the expression of cytokines in murine or human Th17 cells and effectively reduced EAE severity in mice [73]. Therefore, SR1001 and its derivatives may represent a novel drug to treat not only Th17-mediated autoimmune diseases, but ROR-mediated metabolic diseases as well.

By modifying the SR1001 scaffold, SR2211 was developed. Unlike SR1001, SR2211 can specifically inhibit the transcriptional activity of ROR*γ*, but not ROR*α*. In cotransfection assays, SR2211 suppresses transcription activity in both GAL4-ROR*γ* LBD and full-length ROR*γ* contexts. Furthermore, SR2211 could result in suppression of gene expression and production of IL-17 in EL-4 cells [74]. These data strongly suggest that SR2211 is also a potent and efficacious ROR*γ* mediator and represses its activity. Moreover, SR2211 suppressed inflammatory T cell function and Th17 cell differentiation and markedly reduced joint inflammation in mice with CIA [75]. It is shown that SR2211 has the potential utility for the treatment of Th17-mediated autoimmune disorders.

### 4.4. Ursolic Acid

Ursolic acid (UA), a small molecule present in herbal medicine, was identified by screening a small chemical library. In treatment with UA, the function of ROR*γ*t was inhibited selectively and effectively, and IL-17 expression was greatly decreased in developing and differentiated Th17 cells. In addition, UA ameliorated EAE in mice. The results thus indicate that UA might be a valuable drug candidate and can be used for developing treatments of Th17-mediated inflammatory diseases [76].

### 4.5. TM920, TMP778, and GSK805

Using a fluorescence resonance energy transfer (FRET) assay and two-cell line reporter assay (IL-17F promoter and ROR*γ*-LBD promoter assays), a proprietary small-molecule library was screened and several compounds binding to ROR*γ*t were identified. TM920 and TM778 were identified as highly potent and selective ROR*γ*t inhibitors [77, 78].* In vitro*, TM920 and TM778 suppressed Th17 development and inhibited IL-17 production from differentiated Th17 cells. Furthermore, TMP778 has increased potency and specificity for Th17 differentiation, resulting in blockade of nearly all Th17 signature gene expression [77]. Importantly, TMP778 displays no activity against any of the other 24 nuclear receptors tested, including ROR*α* and ROR*β*, so TMP778 has very limited effects on the expression of other genes [78]. TMP778 potently impaired the IL-17 production not only by human CD4^+^ Th17 cells, but also by human CD8^+^ Tc17 cells, memory CD4^+^ T cells, and PBMCs. TMP778 also blocked IL-17 production by skin mononuclear cells of psoriasis patients and significantly impaired expression of Th17 signature gene from psoriasis patients [78].* In vivo,* TMP778 suppressed imiquimod-induced cutaneous inflammation and EAE [77, 78]. Although the specific ROR*γ*t inverse agonist, TM778, may have good ROR*γ*t target effects and low off-target effects, unexpected toxicity may occur in nonimmune cells and tissues (see below); in particular, it required a relatively higher dose of TN778 to exert its function. Another ROR*γ*t inhibitor, GSK805, is proved to be more potent than TM778 and can be orally administered. GSK805 could efficiently ameliorate the severity of EAE and strongly inhibited Th17 cell differentiation in the central nervous system [77]. It is interesting but unexpected that TMP778 and GSK805 were able to induce ROR*γ*t biding to GATA3 and led to an increase of GATA3 mRNA and protein expression. The apparent transactivation of GATA3 by ROR*γ*t may partially explain the inhibition of Th17 cell signature gene expression by TMP778 or GSK805 [77].

These compounds target ROR*γ*t, which inhibit the transcriptional activity of ROR*γ*t by binding to ROR*γ*t LBD [79], a domain present in both ROR*γ* and ROR*γ*t. These compounds not only inhibit Th17 cell differentiation and IL-17 production, but also have shown variable levels of efficacy in EAE and CIA studies. Therefore, these compounds may serve as novel attractive drugs to treat Th17-mediated autoimmune disorders. However, we should note that ROR*γ* is broadly expressed in many human tissues such as heart, kidney, liver, lung, brain, and muscle, so ROR*γ*/ROR*γ*t inverse agonists might induce toxicity via inhabitation of ROR*γ* in nonimmune tissue. Thus, in order to treat Th17-mediated autoimmune disorders, it is necessary to develop a specific strategy to only inhibit ROR*γ*/ROR*γ*t transcriptional activity in immune cells, especially CD4^+^ T cells.

## 5. Targeting Th17 Cells by CD4 Aptamer-ROR*γ*t shRNA Chimera

Recently, RNA interference (RNAi) technology provides a promise for studying basic T cell biology and for developing potential T cell targeted therapeutics. However, efficient delivery of small interference RNA (siRNA) into primary T cells remains a major hurdle of siRNA-based therapy [80]. Emergence of CD4 aptamers, which specifically bind CD4^+^ T cells and efficiently deliver various biomolecules into these cells, makes it possible to target ROR*γ*t and IL-17 production in CD4^+^ Th17 cells with RNAi technology. Here we will discuss the advantage of aptamer-siRNA and contemplate whether CD4 aptamer-ROR*γ*t shRNA chimeras would be beneficial to inhibit Th17 differentiation in human T cells.

### 5.1. Aptamers

Aptamers, nucleic acid-based ligands, are small single-stranded DNA or RNA oligonucleotides that are produced* in vitro* via a process known as systematic evolution of ligands by exponential enrichment (SELEX) [81, 82]. In the SELEX process, aptamers are selected from a large pool (>1 × 10^14^) of single-stranded oligonucleotides with random sequences [83, 84]. After the incubation of the random aptamers pool with the target, followed by repeated cycles: the fixation of region containing binding, PCR or RT-PCR amplification, and modification of restriction endonuclease, aptamers with high affinity with their corresponding ligands are cloned [85]. With the technological improvement in the SELEX process, researchers can isolate aptamers from not only a protein target but also a complex mixture including cell-surface proteins and human plasma in the past decades. Recently, isolation of cell- and receptor-specific aptamers using living cells has been reported [86–88]. Therefore, the power of SELEX enables one to generate specific aptamers against a molecule, a protein, a cell-surface receptor, and even a cell [89, 90]. Notably, chemical modifications to aptamers, including sugar modifications (2′-O-Methyl, 2′-O-methoxyethyl, 2′-fluoro, or LNA), the phosphate backbone modifications (phosphorothioate, boranophosphate), or the nucleobase moiety modifications (4-thiouracil, 2-thiouracil, and diaminopurine), have been reported to greatly enhance the nuclease resistance of the aptamer probes [91, 92].

Similar to antibodies, aptamers, which are often regarded as nucleic acid “antibodies,” gain entrance to target cells via receptor-mediated endocytosis upon binding to cell surface ligands [93, 94]. However, aptamers are generally nonimmunogenic or low-immunogenic [95, 96], whereas antibodies suffer from immunogenicity, resulting in immune responses in patients [97]. In addition, the cost of generation of aptamers* in vitro* is much less than the development process of antibodies [93, 98]. Importantly, aptamers can be generated through simple chemical approach in animals or cultured mammalian cells, making them easier to produce for large scale manufacturing that are necessary for clinical use [99]. The first therapeutic aptamer, antivascular endothelial growth factor (VEGF) aptamer, Macugen (pegaptanib) was approved by the US FDA for treatment of age related macular degeneration in 2005 [100]. Also, several aptamers are currently undergoing clinical trials [92, 101, 102]. Therefore, aptamers that target various proteins and cells are considered as ideal diagnostic and therapeutic approach for clinical disease, such as cancer, infection, and autoimmune disease [99, 103].

### 5.2. Aptamer-siRNA

RNAi offers a powerful approach to developing new therapeutics in human diseases [105]. siRNA, because of their ability to silence expression of sequence-specific gene [106, 107], has currently been developed as a new strategy in treatment of human disease. However, it is a big challenge to efficiently and safely deliver siRNA into “difficult-to-transfect” primary T cells by conventional transfection methods. For instance, electroporation and nucleofection cause excessive cell death and may require preactivation of T cells and electrical apparatus [108, 109]. Chemically modified synthetic siRNA with Acell agents can be transfected into primary T cells; however, they are needed to incubate with T cells for longer time and only a small portion of T cells are transfected [110]. The most disappointing defect of these methods is that it is difficult for them to be used* in vivo*. Retroviral vectors carrying shRNA cassette are able to effectively infect and enter T cells and make the shRNA to stably be expressed for the lifetime of the cells* in vitro* and* in vivo* [111, 112]. However, applying retroviral vectors* in vivo* gives rise to the danger about malignant transformation, which limits the viral vector transfection [80]. Nanoparticles are effective to deliver siRNA into T cells, but the delivery is not T cell specific [113]. Recently, a method that uses a fusion protein composed of a cell-target antibody fragment joined to a protamine peptide that binds nucleic acids has been reported for cell-specific siRNA transfection of immune cells [114, 115]. siRNAs mixed with the fusion protein can silence gene expression in cells, both* in vitro* and in tissues. Modifications of this approach effectively inhibit HIV infection in humanized mice [116]. However, antibody-based fusion proteins are expensive to manufacture, are potentially immunogenic, and are unsuitable for clinical use. Hence, an effective siRNA delivery system* in vivo* for targeting T cells has to be developed for treatment of T cells-mediated human disease. Because aptamers can enter target cells via endocytosis and maintain stability after endocytosis, aptamers have been developed as guiding moieties for both drug delivery and nucleic acid transport vehicles such as siRNA and shRNA [117]. Aptamer siRNA chimeras, composed of an siRNA/shRNA fused to an aptamer, provide an attractive alternative for* in vitro* and* in vivo* gene knockdown [118]. The aptamer portion of the chimeras binds to a cell-surface receptor such as prostate surface membrane Ag (PSMA), CD4, whereas the siRNA portion targets the overexpressed signaling molecules or regulatory nucleic acids, resulting in inhibition of cell proliferation and differentiation. Aptamer-siRNA chimeras (AsiCs) efficiently transfect and knock down gene expression in cells bearing the surface receptor recognized by the aptamer. The PSMA aptamer-siRNA chimeras targeting PSMA silenced target gene expression in prostate cancer mouse xenografts [96]. AsiCs containing an aptamer targeting HIV-gp120 inhibit HIV replication in already infected cells* in vitro* [119, 120] and* in vivo* [121]. CD4-AsiCs bearing siRNAs that recognized HIV* gag* and* vif* or host* CCR5* were specifically taken up by CD4^+^ cells, knocked down genes expression, and inhibited HIV infection in primary CD4^+^ T cells and in the female genital tract of humanized mice [122, 123] and at the same time do not activate lymphocytes or stimulate innate immunity [122, 123]. Moreover, the chimeras do not bind to or function in cells that do not express CD4, such as CD3, CD8, and CD45 [124]. Thus, aptamer-facilitated cell specific delivery of siRNA/shRNA represents an attractive novel approach for efficient RNAi delivery. CD4-AsiCs overcome the hurdle of* in vivo* siRNA delivery to the immune cells and hold a promise to study immune responses and develop therapeutics in autoimmune diseases.

### 5.3. CD4 Aptamer-ROR*γ*t shRNA Chimeras

**Figure 1:**
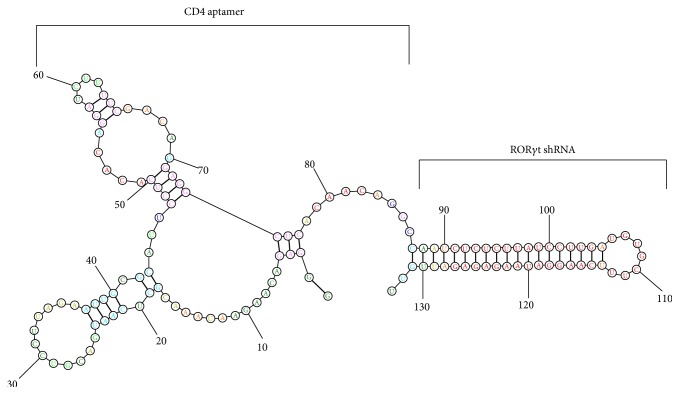
Predicted secondary structure of CD4-aptamer-ROR*γ*t shRNA chimera (modified from Song et al., BBRC, 2014, Figure  1(b) [104] with permission).

CD4 aptamers that bind surface CD4 can be applied as T helper cell-specific delivering vehicles. CD4-AsiCs bearing siRNAs or shRNA targeting ROR*γ*t might suppress Th17 differentiation and treat Th17-mediated autoimmune diseases. We selected CD4 RNA aptamers (86 nucleotides in length) that delivered ROR*γ*t-shRNA (Figure 1) into CD4^+^ T cells and investigated its efficacy in suppressing Th17 cell differentiation and IL-17 production in human CD4^+^ T cells* in vitro* [104]. Chemical modifications of nucleotides* 2*′*-F-dCTP and 2*′*-F-dUTP* were done to enhance the nuclease resistance of the aptamer chimeras [104].* In vitro* using fluorescent microscope and flow cytometric analysis, Cy3-labeled CD4 aptamer-ROR*γ*t shRNA chimeras (CD4-AshR-ROR*γ*t) (133 nucleotides in length) were shown to enter into human CD4^+^ T cells but not Cy3-labeled mock CD4-AshR- ROR*γ*t.* In vitro* expression of ROR*γ*t is significantly and specifically diminished by CD4-AshR-ROR*γ*t in a concentration-dependent manner in human CD4^+^ T cells compared with control CD4 aptamers [104]. Consistent with decreased ROR*γ*t, CD4-AshR-ROR*γ*t displayed a concentration-dependent inhibition of IL-17A release from CD4^+^ T cells and intracellular IL-17A staining in CD4^+^ T cells, while mock CD4-AshR-ROR*γ*t and CD4-AshR-scrambled control have no impacts [104]. This study indicates that intracellular delivery of CD4-AshR-ROR*γ*t could target ROR*γ*t and manipulate Th17 cell differentiation and IL-17 production in CD4^+^ T cells. Additionally, CD4-AshR-ROR*γ*t does not significantly impact the expression of Th1 and Th2 lineage transcription factors T-bet and GATA-3 in PMBCs. Consistent with these, synthesis of IFN-*γ* and IL-4 in PBMCs is not changed by CD4-AshR-ROR*γ*t.

These suggest that CD4-AshR-ROR*γ*t chimeras keep its specificity to target ROR*γ*t gene and Th17 cells. Thus, it is of interest to explore the use of CD4-AshR-ROR*γ*t chimeras in animal and clinical trials of autoimmune diseases.

## 6. Concluding Remarks

In conclusion, Th17 cells and their signature cytokines play crucial roles in the pathology of autoimmune and inflammatory diseases. Targeting IL-17 or IL-17R has shown clinical efficacy in psoriasis but not many other autoimmune disease such as RA and Crohn's disease. In contrast to blocking a single effector cytokine, targeting Th17 lineage provides promising therapeutic to impact multiple inflammatory cytokines. First attempts to target Th17 lineage are targeting ROR*γ*t, the master transcriptional factor of Th17 lineage, via small molecule inverse agonists. Several small molecules are shown to have potent suppressive effects on Th17 cells and their cytokines and have therapeutic efficacy in animal models of autoimmune diseases. Clinical studies are required to assess their usefulness for treating Th17-mediated human diseases. Aptamer mediated delivery of siRNA/shRNA specifically against ROR*γ*t offers another strategy to target Th17 cells. By replacing the shRNA for targeted genes such as GATA3, T-bet, and STAT3; this CD4 aptamer may be used as a universal tool to introduce siRNA or shRNA into CD4^+^ T cells to manipulate function of various Th cells. Further animal and clinical trials of CD4-AshR-ROR*γ*t chimeras are necessary to evaluate the beneficial outcomes in autoimmune diseases.
